# *Toxoplasma gondii* in small mammals in Romania: the influence of host, season and sampling location

**DOI:** 10.1186/s12917-023-03729-7

**Published:** 2023-09-29

**Authors:** Zsuzsa Kalmár, Attila D. Sándor, Anamaria Balea, Silvia-Diana Borşan, Ioana Adriana Matei, Angela Monica Ionică, Călin Mircea Gherman, Andrei Daniel Mihalca, Anamaria Cozma-Petruț, Viorica Mircean, Adriana Györke

**Affiliations:** 1 University of Agricultural Sciences and Veterinary Medicine of Cluj-Napoca, Cluj- Napoca, RO-400372 Romania; 2 “Iuliu Hațieganu“ University of Medicine and Pharmacy, Cluj-Napoca, Romania; 3 HUN-REN-UVMB Climate Change: New Blood-sucking Parasites and Vector-borne Pathogens Research Group, Budapest, Hungary; 4 Department of Parasitology and Zoology, University of Veterinary Medicine, Budapest, Hungary; 5 Sanitary Veterinary and Food Safety Directorate Cluj, Cluj-Napoca, Romania; 6 Clinical Hospital of Infectious Diseases of Cluj-Napoca, Cluj-Napoca, Romania

**Keywords:** Epidemiology, Felinae, Rodents, protozoa, Synanthropic rodents

## Abstract

**Background:**

*Toxoplasma gondii* is a protozoan parasite that infects a large spectrum of warm-blooded animals, including humans. Small rodents and insectivores play an important role in the epidemiology of *T. gondii* and may serve as a source of infection for both, domestic and wild definitive felid hosts. Factors influencing the occurrence of *T. gondii* in wild small mammals are unknown, despite the fact that many intermediate host species are identified. We have used small mammals (Rodentia and Lipotyphla) captured over two years in various habitats, both in urbanised and in natural landscapes. We assessed the importance of land-use, season and host ecology on *T. gondii* infection.

**Results:**

We examined 471 individuals belonging to 20 small mammal species, collected at 63 locations spread over wide altitude, habitat and land-use ranges from Romania. Heart tissue samples were individually analysed by PCR targeting the 529 bp repetitive DNA fragment of *T. gondii*. The overall prevalence of infection was 7.3%, with nine species of rodents and two species of shrews being found to carry *T. gondii* DNA. Five species showed high frequency of infection, with the highest prevalence found in *Myodes glareolus* (35.5%), followed by *Spermophilus citellus* (33.3%), *Sorex minutus* (23.1%), *S. araneus* (21.7%) and *Micromys minutus* (11.1%). Adults seemed more often infected than young, however when controlling for season, the difference was not significant, as in spring both adults and young showed higher infection rates, but more adults were sampled. Contrary to our expectations, urban/rural areas (with their implicit high density of domestic feline presence) had no effect on infection prevalence. In addition, neither habitat, nor land-use at sampling sites was important as only geographical location and host species were contributing factors to the infection risk.

**Conclusions:**

High prevalence of *T. gondii* infection showed a highly localised, patchy occurrence, with long living and higher mobility host species being the most common carriers, especially during autumn.

**Supplementary Information:**

The online version contains supplementary material available at 10.1186/s12917-023-03729-7.

## Background

Toxoplasmosis is an infection caused by *Toxoplasma gondii*, an apicomplexan zoonotic parasite with a world-wide distribution, that may infect a wide range of warm-blooded vertebrates [1]. Toxoplasmosis is one of the most prevalent zoonotic diseases worldwide, and ranks among the food-borne illnesses with high disease burden [2, 3]. About one third of the human population is estimated to be infected with this parasite, posing important risks especially for pregnant women and immunocompromised patients [4]. Although toxoplasmosis in wildlife has little clinical impact, there are certain animal species (pigeons, canaries) that can be severely affected [5]. *Toxoplasma gondii* may also be responsible for considerable economic losses in farming systems, as the parasite may be pathogenic to livestock [6].

Members of the *Felidae* family serve as definitive hosts of *T. gondii* and are the only hosts that shed *T. gondii* oocysts into the environment [7, 8]. Transmission of the parasite can occur through three main routes: (1) ingestion of *T. gondii* oocysts eliminated in the faeces of felids, with contaminated water or food; (2) consumption of raw or undercooked meat or organs containing viable tissue cysts; or (3) congenitally, by transplacental transmission of the parasite after maternal infection acquired during gestation [2]. The life cycle of *T. gondii* includes a sylvatic transmission in most natural habitats (definitive hosts are the wild felids), and a domestic transmission cycle inside anthropised environments, where the definitive host is the domestic cat. These two types of transmission cycles may co-occur in areas where domestic/feral cats roam outside human settlements [9]. This parasite has a broad range of intermediate hosts such as birds, mammals (including humans and small mammals), but also invertebrates [10].

Several small mammals are keystone species and represent a major component of predators’ diets (especially for wild and domestic felids), thus having an important role in the ecological food chain [11–13]. Domestic felids are particularly interesting since these are possible reservoirs for the parasite, but also may represent a direct source of infection for humans. Therefore, understanding the relationships between small mammals and *Toxoplasma* prevalence is of great importance for the management of small mammal populations from both conservation and control points of view [13].

The independent existence and importance of the domestic and sylvatic cycles is currently widely debated [14], and most authors agree that studies targeting multiple potential wildlife intermediate hosts are needed [11, 15]. Nevertheless, there are many aspects of the transmission cycle which may have a potential for confounding effects. Local climate, soil, land-use, or habitat composition determine small mammal species composition, abundance, and reproductive cycles [16]. However, these environmental conditions may also act differentially on *T. gondii*-infection risk for intermediate hosts, for example through the direct impact of different temperature and humidity ranges on oocyst survival [9, 17]. To study the possible effects of complex environments on the transmission cycle of *T. gondii*, we collected data on *T. gondii* infection status in small mammals along a wide range of elevations (0-2000 m), diverse habitats and seasonal occurrences in Romania. Romania hosts five ecoregions on its territory, thus having a high plant and animal diversity, which includes up to 52 small mammal (Rodentia and Lipotyphla) species [18]. Although small mammal and tick-borne pathogen associations [19–21], as well as toxoplasmosis have been previously investigated in livestock (cats, pigs, horse, goats, broiler chickens, calves, sheep, cattle), wild hosts (red foxes, wild boar) and humans in Romania [22–30], the prevalence of *T. gondii* in small mammals is still unknown. Moreover, we also lack information on the potential impact of environmental conditions (e.g. climate, elevation, land-use) or local risk factors (abundance of suitable oocyst-shedders) on infection levels. In this context, the aim of the present study was to provide epidemiological data from a survey on the presence of *T. gondii* infection in small mammals from Romania.

## Results

Altogether, heart samples of 471 individuals were analysed. These represented 20 species, most being rodents (364 individuals, 14 species, see Suppl File. nr.1), with an additional 107 insectivores (the rest of the species). Mice (Muridae) made up nearly half of all captures (n = 233, 49.5%), followed by voles (Cricetidae, n = 125, 26.5%) and shrews (Soricidae, n = 96, 20.4%). *Apodemus agrarius* was the most frequently sampled species (n = 82, 17.4%), followed by *Mus musculus* (n = 54, 11.5%) and *Microtus arvalis* (n = 47, 10%), the rest of species did not exceed 5% (Table 1). The sex ratio of the samples was slightly female biased (0.88); adults represented 37.6% and yearlings were 62.4% of all individuals of known age. Most individuals were collected in grasslands (46.0%), but considerable numbers were from forests (30.1%) and inbuilt areas (19.1%), too. Urbanisation gradient was representative, with most samples collected from natural areas (n = 211, 44.8%), followed by rural/farmland areas (n = 202, 42.9%) and urban ones (n = 58, 12.3%).

**Table 1:** Infection prevalence of *Toxoplasma gondii* in tissues of small mammal species (N – total number of samples; CI: 95% confidence interval)

Species	*N*	Positive	Prevalence (%)	CI
*Apodemus agrarius*	82	1	1.2	0.1–6.6
*Apodemus flavicollis*	47	4	8.5	2.4–20.4
*Apodemus sylvaticus*	22	2	9.1	1.1–29.2
*Apodemus uralensis*	3	0	0	-
*Arvicola amphibious*	1	0	0	-
*Crocidura leucodon*	19	0	0	-
*Crocidura suaveolens*	40	0	0	-
*Micromys minutus*	9	1	11.1	0.3–48.3
*Microtus arvalis*	47	2	4.3	0.5–14.5
*Microtus subterraneus*	46	2	4.4	0.5–14.8
*Mus musculus*	53	3	5.7	1.2–15.7
*Mus spicilegus*	2	0	0	-
*Myodes glareolus*	31	11	35.5	19.2–54.6
*Neomys anomalus*	1	0	0	-
*Ondatra zibethicus*	3	0	0	-
*Rattus norvegicus*	15	0	0	-
*Sorex araneus*	23	5	21.7	7.5–43.7
*Sorex minutus*	13	3	23.1	5.0-53.8
*Spermophilus citellus*	3	1	33.3	0.8–90.6
*Talpa europaea*	11	0	0	-
TOTAL	471	35	7.4	5.4–10.2

The overall *T. gondii* prevalence in micromammal tissues was 7.4% (35/471; 95% CI: 5.4–10.2). From the total of 20 small mammal species, 11 species (55.0%; 95% CI: 31.5–76.9) harboured *T. gondii* DNA, with considerable differences in prevalence. As a taxonomic group, voles (Cricetidae) showed the highest prevalence (12.0%), followed by shrews (8.3%) and mice (5.1%). The highest prevalence was identified in *Myodes glareolus* (35.5%, 11 out of 31), followed by *Spermophilus citellus* (33.3%, 1 out of 3), *Sorex minutus* (23.1%, 3 out of 13), *S. araneus* (21.7%, 5 out of 23), and *Micromys minutus* (11.1%, 1 out of 9). For the infection status of individual species please refer to Table 1. The infection prevalence was significantly different between small mammal species (*χ*^2^ = 63.9995, *df* = 19, *P* < 0.01).

When all data was compared, we found differences in *T. gondii* prevalence between different age categories, with more adults being infected (16.1% vs. 8.7%), although this difference was significant only in autumn. Significantly more individuals were infected in spring (independent of age), compared to any other season (Table 2.).

**Table 2:** Effect of different predictors on the presence and prevalence of *Toxoplasma gondii* in small mammal tissues (GLM, n = 471, CI: 95% confidence interval, significant predictors in bold and noted with *)

Predictors	Odds Ratios	CI	*p*
*(Intercept)*	*0.16*	*0.01–2.94*	*0.226*
**Host species**	**0.75**	**0.57–1.01**	0.049*
Host age	0.71	0.34–1.51	0.370
Host sex	0.97	0.53–1.68	0.912
**Season**	**0.47**	**0.25–0.90**	0.020*
**Locality**	**1.03**	**1.00–1.07**	0.029*
Land use	3.22	0.34–25.32	0.281
Elevation	1.00	1.00–1.00	0.784

Although more infected hosts were found in forested areas and in the alpine region (above 1200 m a.s.l.), however, only season (Z = 0.0982) was retained as a significant predictor of *T. gondii* positivity when the combined effect of multiple predictors was tested (logistic regression, sampling location used as a random effect). The GLM only identified the season, host species, or capture location as important contributing factors to *T. gondii* infection (Table 2.).

## Discussion

As small mammals may play a substantial role in the transmission of *T. gondii* to felids, they may spread the infection in the environment by increasing the risk of human infection [10]. Therefore, the aim of the present study was to evaluate the importance of host ecology, habitat and location on the prevalence of *T. gondii* in small mammals in Romania. These results have shown variable prevalence of *T. gondii* in different small mammal species, with a wide variety of hosts (11 species). *Toxoplasma gondii-*prevalence values recorded in Europe present a high variability, ranging between 1 and 90% in different mammalian host populations [31]. The overall prevalence was 7.4%, which lays in the middle range for most reports in small mammals [32]. A global meta-analysis which assessed the seroprevalence of *T. gondii* in small mammals from 1970 to 2018, reported a seroprevalence of 6% of anti-*T. gondii* IgG antibodies, varying between continents from 24% in Africa to 18% in South America and even 1% in Europe [10]. Although this study is based on serology, its results are important, because even a low prevalence in small mammals may build-up a high level of infection in felids, as they consume many small mammals throughout their lives.

Diurnal and long-living squirrels are key species for the epidemiological cycle of *T. gondii* [33]. Here, despite the low number of squirrels tested (*n* = 3), one animal was positive resulting in a high infection rate. Although European sousliks (*S. citellus*) have a patchy distribution, they may be locally important hosts in the transmission of *T. gondii*, as feral cats are important predators of sousliks [34]. Experimental studies on the reservoir role of different squirrel species already prove that certain species (*Citellus tridecemlineatus*, *Petaurista petaurista grandis*, *Sciurus carolinensis*, *S. vulgaris*, *Tamias sciurus hudsonicus* and *T. sibericus*) are susceptible to *T. gondii* infection [33, 35]. In a survey performed in Slovakia [36], an infection prevalence of 16.6% was reported for the red squirrel (*S. vulgaris*), in addition Kik et al. [33] found that *T. gondii* infection was the likely cause of death for several red squirrels in the Netherlands. Although serological evidence is available for a number of different squirrel species, this is the first report of *T. gondii* DNA in *S. citellus*. As the number of the investigated squirrels was low in the current study, it is not possible to conclude the importance of European sousliks in the enzootic cycle of *T. gondii*.

In the present study, the highest prevalence among voles was recorded in bank voles (*M. glareolus*, 35.5%), while only two (4.4%, n = 46) European pine voles (*Microtus subterraneus*) were positive for *T. gondii*. Voles are regular hosts of *T. gondii*, with highly variable prevalence rates reported. Infection was already proven for voles in the Netherlands [37] or Slovakia [38], and a 17% prevalence was reported for this group in Austria [39]. We found a relatively high prevalence (8.3%) of *T. gondii* infection in shrews (21.7% for *S. araneus* and 23.1% for *S. minutus*). Shrews were already mentioned as *T. gondii* hosts; Kijlstra et al. [40] reported a prevalence of 13.6% for the white toothed shrew (*Crocidura russula*) in the Netherlands.

Different mice species showed contrasting results in *T. gondii* infection in our study. While all host species which were sampled in higher numbers (n > 20) included positive samples (mean 5.1, range 1.2–11.1), both forest dwelling as well grassland specialists showed fluctuating ranges (Table 1). The role of *A. flavicollis* and *A. sylvaticus*, the most common mouse species in Europe [41], is well acknowledged in the circulation of *T. gondii*. Interestingly, in our study Eurasian harvest mouse (*M. minutus*) showed the highest prevalence of *T. gondii* infection, a phenomenon rarely observed before [17, 40, 42]. Low infection prevalence was found in *A. agrarius* (1.2%), the most common and widespread mouse species collected (n = 82, 24 locations). This is in line with several studies all over the species’ range, where low prevalence is the norm (44,45).

Several studies reflect implication of the house mice (*M. musculus*) and brown rat (*R. norvegicus*) in the transmission cycle of *T. gondii* [12]. Both are commensal species living in close proximity to humans, sharing their biotopes with domestic cats [12, 43]. Due to the activity of specific macrophages, rats are more resistant to *T*. *gondii* infection than mice and have a different ecology compared to other small mammal species [44]. Dubey and Frenkel [45], summarized the worldwide prevalence of *T*. *gondii* in different species of rats and concluded that the prevalence of viable parasites in *R*. *norvegicus* was generally low. In contrast, high prevalence was reported in a study where *M. musculus* and *R. norvegicus* were captured within domestic dwellings in England as part of a pest control programme. The PCR analysis revealed that 53% of *M. musculus* and 42.2% of *R. norvegicus* were *T. gondii* positive [46]. Kijlstra et al. [40], reported an overall prevalence of 11.9% in small mammal species, respectively 10.3% in *R. norvegicus*, 6.5% in *M. musculus* and 14.3% in *A. sylvaticus*. Although certain studies reported fairly high prevalence rates for these two commensals [12, 31, 46–48], we failed to detect *T. gondii* in *R. norvegicus*, and found it in very few *M. musculus* (5.7%).

We found major differences in prevalence according to season and host age, with adult hosts showing higher prevalence scores. Similar results were already reported from France [43] and Poland [49]. Seasonal differences may be attributable to more favourable climatic conditions for the survival of *T. gondii* oocysts in spring. It is known that oocyst survival is highly dependent on local humidity [9], which is highest in spring in Romania (compared to summer or autumn) [50]. Another factor that may favour higher prevalence rates in boreal spring is the higher age ratio of adults in the population in comparison to late summer/autumn [16]. Likewise, age per se may contribute to higher infection risk, as throughout their life, small mammals may accumulate infection, through higher mobility in the dispersion period, when young adults travel distances greater than their species-specific home-range, thus increasing their chance to encounter an infection source [16]. Although different habitats and land-use categories may predict local small mammals’ abundance and species composition, we found no effect of habitat, land-use or even elevation on *T. gondii* infection levels, likely because infection risk is dependent mainly on the presence/absence of an active spreader (felids shedding oocysts) and local climatic optimum (oocyst survival). While other infection routes were also proposed for small mammals, like congenital/vertical [31, 51], the fact that at certain locations multiple species were positive for *T. gondii* DNA (e.g. if we used sampling location as a random effect, host species was not defining) indicate locally important horizontal infection-source. Proximity of human inhabitation was proposed as an important factor for *T. gondii* infection risk, due to the high density of the potential definitive hosts, domestic/feral cats. Animals in urban environments may be highly exposed to the parasite, thus showing a higher prevalence as compared to those from rural areas, due to the habitats they live in, and the different degree of contamination with oocyst from felids [17, 43, 51, 52]. In the present study, we failed to prove this, as we found no significant differences between *T. gondii* infection prevalence of small mammals caught at sites belonging to different urbanisation gradients.

Our analyses revealed that apart from season and host species characteristics, only location was an important predictor of *T. gondii* absence/presence, as well as prevalence in local small mammal populations. However, when sampling location was used as a random effect, not even host species predicted *T. gondii* infection risk. Sampling site was important independent of its habitat, land-use or its proximity to an anthropic area (likely high density of potential *T. gondii* definitive hosts), showing that neither the physical environment, nor small mammal host species determines *T. gondii* infection risk, but the season and the (likely) presence of a successful *T. gondii* infection-source.

## Conclusions

The results of this study confirm the infection with *T. gondii* among small mammals (*S. citellus*, *M. glareolus*, *S. araneus, S. minutus*, and *M. minutus*) in Romania, that may potentially play a role as reservoirs of the parasite. To our knowledge, the present study is the first to evaluate the occurrence of *T. gondii* DNA in small mammals from Romania, highlighting the importance of wild animal surveillance for toxoplasmosis. Our analysis showed that neither sampling habitat, nor local land-use was important, whereas sampling location and host species were the main contributing factors to infection risk. Higher prevalence of *T. gondii* infection showed a highly localised, patchy occurrence, with long living and higher mobility host species being the most common carriers, especially in spring.

## Methods

### Sample collection

**Fig. 1:**
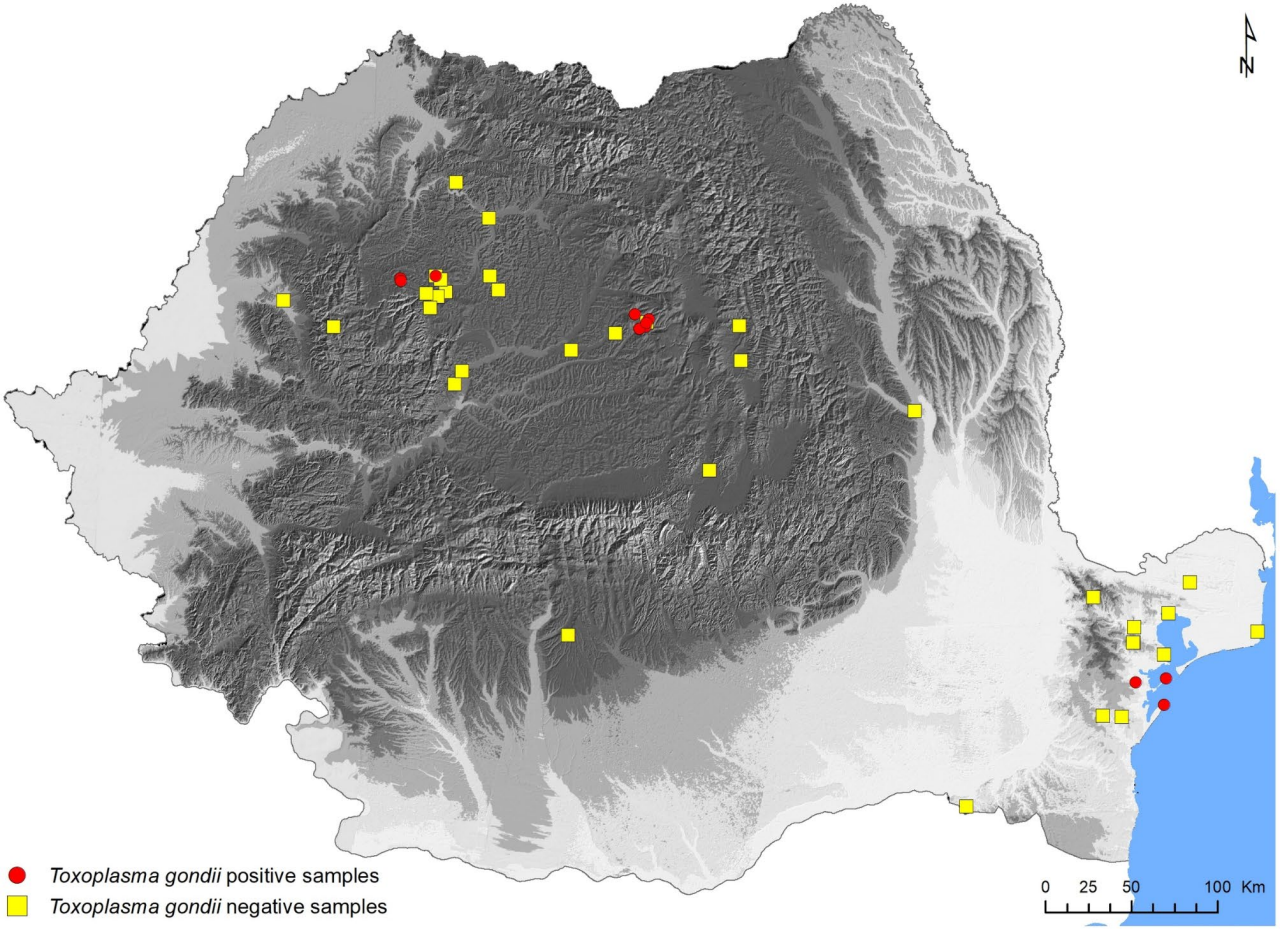
Map with the location of sampling grids for capturing small mammals, with presence/absence of *Toxoplasma gondii* in mammal tissues (original)

Small mammals were collected during 2010–2011 in natural and anthropic habitats in Romania (Fig. 1). Most samples used in this study were collected from small mammals caught using commercially available humane-kill snap traps (completed with samples of fresh road kills in the case of protected species). Baited snap traps were erected in distinct capture stations (suitable patches of vegetation with grids of 50 traps each), and controlled every six hours for 48 h. Capture stations were allocated to different land use (wetland, grassland, arable, forest, urban), elevation gradient (lowland, hills, upland and alpine), urbanisation gradient (natural, rural, urban) and seasonal categories. We determined three levels of urbanisation: natural areas (any site laying a distance of more than 2 km from the closest farm or human habitation, a conservative estimate, to exceed the maximum known home-range of up to 7.4 km^2^ for feral cats [53], rural (any site with semi-natural vegetation which lays inside/close to farmed landscape, but away of urban centres) and urban (built-in areas, inside towns and cities, trapped species exclusively commensals). Trapped mammals were marked locally and individually stored in zip-lock bags at temperatures below − 20 °C until processing. Detailed methods used for species identification, ageing, sexing and tissue collection were described elsewhere [18, 19]. We used the heart tissue (25 mg) in order to screen for the *T. gondii* infection.

### DNA extraction

Genetic material isolation was performed individually from all heart samples using a commercial DNA extraction kit (ISOLATE II Genomic DNA Kit, Meridian Bioscience, London, UK) according to the manufacturer’s instructions. During the DNA extraction procedure, negative controls were used in order to identify possible cross-contamination. DNA samples were stored at -20ºC for further analysis.

### PCR

The DNA samples were assessed by PCR reactions targeting the 529 bp repetitive DNA in *T. gondii* using specific primers Tox4 (5’- CGCTGCAGGGAGGAAGACGAAAGTTG-3’) and Tox5 (5’-CGCTGCAGACACA GTGCATCTGGATT-3’) [54]. PCR was performed in T100™ Thermal Cycle (Bio-Rad, London, UK) using MyTaq Red Mix (Bioline, London, UK) in a final reaction volume of 25 µl. Positive (*T. gondii* RH strain) and negative (water) controls were used during the PCR. Amplicons were visualized by electrophoresis in 1.5% agarose gel stained with SYBR Safe DNA gel stain (Invitrogen, Waltham, MA, USA).

### Statistical analyses

Prevalence (percentage indicating number of infected specimens relative to total studied specimens), and its 95% confidence interval (CI) were calculated in R, v.4.0.5, an open-access environment for statistical computing [55]. To compare parasite prevalence rates, we used Chi-squared (or Fisher’s exact tests). Here we would like to add, that sample size showed wide variation among the different mammal species due to the methodology used (e.g. species specific trapping success, use of road-kill specimens). As a consequence, low sample size may cause bias (through both zero positives, as well as biased zero interpretation). We tried to reduce this bias through different groupings used in the analyses (i.e. taxonomic, sex, age, season, land-use). To test the importance of certain biotic factors (host species, sex, age) and abiotic factors (land-use type, season, elevation, urbanisation gradient) on the presence versus absence of *T. gondii* infection, we used general linear models (GLM) under the assumption of a binomial distribution (absence/presence), with the built-in *glm* function [55]. To test for co-linearity and combined effects of multiple predictors, we ran a logistic regression, where sampling locality was included as a random effect. Differences were considered significant when *p* < 0.05. Map in Fig. 1 was created using ArcGIS 10.6 (ESRI©, Redlands, CA, USA).

### Electronic supplementary material

Below is the link to the electronic supplementary material.

Supplementary Material 1

## Data Availability

All relevant data are included in the manuscript and the references or are available upon request from the corresponding author.
